# Transcriptome profiling unveils GAP43 regulates ABC transporters and EIF2 signaling in colorectal cancer cells

**DOI:** 10.1186/s12885-020-07728-x

**Published:** 2021-01-05

**Authors:** Xi Chen, Hongjin Wu, Jia Feng, Ying Li, Jiao Lv, Weikai Shi, Weiwei Fan, Li Xiao, Danmeng Sun, Mingfeng Jiang, Ming Shi

**Affiliations:** 1 School of Life Science and Technology, Harbin Institute of Technology, Harbin, 150001 China; 2 Department of Clinical Laboratory, Hangzhou Cancer Hospital, Hangzhou,, 320000 Zhejiang China; 3 The NHC Key Laboratory of Drug Addition Medicine, The First Affiliated Hospital of Kunming Medical University, Kunming, 650000 China; 4 Department of Infectious and Medicine, Heilongjiang Provincial Hospital, Harbin, 150036 China; 5 Department of Pediatrics, Data People’s hospital, Shenmu, 719301 China

**Keywords:** GAP43, Colorectal cancer, RNA-seq, DNA methylation, Transcriptome profiling

## Abstract

**Background:**

The growth- and plasticity-associated protein-43 (GAP43) is biasedly expressed in indigestive system and nervous system. Recent study has shown that GAP43 is responsible for the development of neuronal growth and axonal regeneration in normal nervous tissue, while serves as a specific biomarker of relapsed or refractory neuroblastoma. However, its expression pattern and function in digestive system cancer remains to be clarified.

**Methods:**

In this study, we examined the *GAP43* status with qRT-PCR and bisulfite genomic sequencing in colorectal cancer (CRC). We investigated the effect of overexpressed GAP43 in CRC cells with RNA-seq. The RNA-seq data was analyzed with DAVID and IPA.

**Results:**

*GAP43* was downregulated in CRC compared to the adjacent tissues. DNA methylase inhibitor 5-Aza-CdR treatment could significantly induce *GAP43*, indicated that the silencing of *GAP43* gene in CRC is closely related to DNA methylation. Bisulfite genomic sequencing confirmed the promoter methylation of GAP43 in CRC. To explore the transcriptional alterations by overexpressed GAP43 in CRC, we performed RNA-seq and found that upregulated genes were significantly enriched in the signaling pathways of ABC transporters and ECM-receptor interaction, while downregulated genes were significantly enriched in Ribosome signaling pathway. Further Ingenuity Pathway Analysis (IPA) showed that EIF2 signaling pathway was significantly repressed by overexpression of GAP43.

**Conclusion:**

Our findings provide a novel mechanistic insight of GAP43 in CRC. Transcriptome profiling of overexpressed GAP43 in CRC uncovered the functional roles of GAP43 in the development of human CRC.

**Supplementary Information:**

The online version contains supplementary material available at 10.1186/s12885-020-07728-x.

## Background

Colorectal cancer (CRC) is one of the most common type of the digestive tract cancers and it ranks the third in the world’s cancer incidence [1]. CRC is mainly caused by genetic factors, chronic inflammation, unhealthy living habits, etc.; the geographical distribution of the disease is quite different, and the incidence of CRC is higher in developed areas [2]. China is a low-incidence area for CRC, but the incidence rate is increasing [3]. As of 2016, the number of new cases of CRC in China has exceeded 270,000 and more than 130,000 deaths [4]. As the third malignancy in China, previous researches showed that CRCs are caused by various genetic and epigenetic changes, including WNT, PI3K-Akt, GPCR, FGFR, MAPK, TGFB, and TP53 signaling pathways [5–7]. To date, the exact cellular and molecular mechanisms leading to CRC have not been systematically evaluated. Further investigation of functional genes in CRC is critical for identifying novel potential targets for CRC detection and precision treatment.

GAP43 was reported as a ‘growth’ or ‘plasticity’ protein, which was responsible for the development of neuronal growth and axonal regeneration [8–10]. Previous studies of GAP43 thus far were focused on the neuron biology. However, the role of GAP43 in human cancers, especially CRC, has not been explored to date. Previously, with the GEPIA database (http://gepia.cancer-pku.cn/), we found that the expression level of GAP43 was significantly downregulated in colorectal cancer tissues (normal versus cancer tissues), including the colon adenocarcinoma (COAD) and rectal adenocarcinoma (READ) (Supplementary Fig. S1a).

Here, we investigated the expression level of GAP43 in CRC cell lines. The mechanism of the silenced *GAP43* gene expression in CRC tissues was further studied with the analysis of its promoter methylation. Furthermore, the transcriptome alteration of GAP43-overexpressed versus control CRC cell line was analyzed with RNA-seq. Taken together, we disclosed the potential functions of GAP43 in the development of human CRC.

## Methods

### Cell culture and transfection

The CRC cell lines HCT116 (Resource number: 3131C0001000700099), SW620 (Resource number: 3131C0001000700101), SW480 (Resource number: 3131C0001000700086), HT-29 (Resource number: 3131C0001000700103), DLD-1 (Resource number: 3131C0001000700134) were cultured in RPM1640 medium supplemented with 10% fetal calf serum and 2 mM L-glutamine. All CRC cell lines used in this study were originally purchased from the Type Culture Collection of the Chinese Academy of Sciences, Shanghai, China. Normal colorectal epithelial cell line NCM460 (Resource number: CVCL_0460, INCELL) was kindly provided by Dr. Ming Liu, Harbin Medical University, China. NCM460 was cultured in DMEM medium supplemented with 10% fetal calf serum and 2 mM L-glutamine. All cell lines were authenticated by short tandem repeats (STR) analysis. All cell lines have been tested for mycoplasma contamination, and no contamination was found. SW620/GAP43 cell (SW620 cell overexpressing GAP43 protein) was generated by stabilizing a GAP43-expression plasmid that confers neomycin resistance in SW620 cell.

### Patient samples

The collection of three paired CRC samples used for quantitative Real-Time PCR, patient consent, and patient recruitment followed Institutional Review Board protocols from Hangzhou Cancer Hospital. The clinical information of these colorectal cancer tissues was listed in Supplementary Table S6. The study has been approved by the Research Ethics Committee of Hangzhou Cancer Hospital (Hangzhou, P. R. China; approval number HZCH-2018-02). All written informed consents have been provided by the patients, and to be conducted in accordance with the Declaration of Helsinki.

### Quantitative real-time PCR (qRT-PCR)

Quantification of mRNA expression was performed with SYBR Green PCR Master Mix on an ABI 7500 real-time PCR system (Foster City, CA, USA). GAPDH was considered as an internal control. The qRT-PCR procedure was recommended according to the protocol. Primers for qRT-PCR are described in Supplementary Table S7.

### Bisulfite DNA sequencing

Bisulfite DNA sequencing was performed as previously described [11]. Briefly, the bisulfite modification of DNA was performed according to the protocol of the methylSEQr™ Bisulfite Conversion Kit (Applied Biosystems, Shanghai, CN). The bisulfite-treated DNA was amplified using nested PCR using the primers described in Table S1. The amplified bisulfite-treated DNA was sequenced and compared to the original sequence of the *GAP43* gene.

### RNA-seq library preparation and sequencing

Total RNA was isolated from 4X10^6^ GAP43 overexpressed and control SW620 cells using RNeasy Mini Kit (Qiagen, Valencia, CA). Total RNA integrity was checked using an Agilent 2100 Bioanalyzer (Agilent Technologies, Inc., CA, USA) with an RNA integrity number (RIN) value greater than 8. The mRNA was purified and fragmented from total RNA (2 μg) using poly-T oligo attached magnetic beads with two rounds of purification. Then the cleaved RNA fragments were primed with random hexamers and were reverse transcribed into first-strand cDNA using reverse transcriptase and random primers. The RNA template was removed and synthesized a replacement strand to generate double-strand cDNA. End repair, A-tailing, adaptor ligation, cDNA template purification and enrichment were then performed. Constructed libraries were sequenced with 150 bp paired-end by an Illumina X10 sequencer, following manufacturer’s instructions.

### Database for annotation, visualization and integrated discovery (DAVID) and ingenuity pathway analysis (IPA)

DAVID (http://david.abcc.ncifcrf.gov), Functional Annotation Bioinformatics Microarray Analysis was used to identify significantly enriched gene ontology (GO) and Kyoto Encyclopedia of Genes and Genomes (KEGG) terms among the given list of genes that are differentially expressed in GAP43 overexpressed and control HCT116 cells. Statistically overrepresented GO and KEGG categories with *p*-value≤0.05 were considered significant. For IPA, different expressional genes (DEGs) were submitted to IPA software (Qiagen bioinformatics, Ingenuity Pathway Analysis), the canonical pathways were considered significant with *p*-value≤0.05 and z-score ≥ 1 or z-score ≤ − 1.

### Statistical analysis

Statistical data were represented as mean ± standard deviation from at least three independent experiments. Genes expression were statistically analyzed by two-way repeated measures ANOVA. All graphs were plotted and analyzed with GraphPad Prism 7 software.

## Results

### GAP43 is expressed in human colon tissues, but not in colorectal cancer cells

**Fig. 1:**
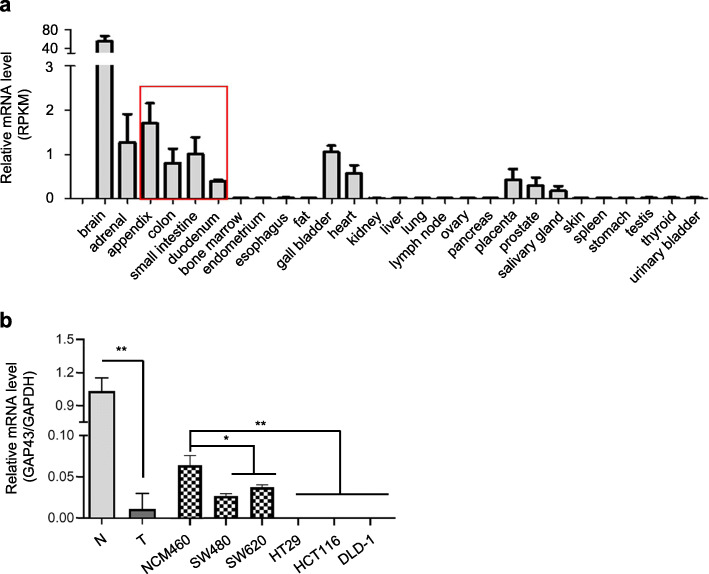
GAP43 expression levels are detected in normal human tissues and colorectal cancer cell lines. **a** Relative mRNA levels are analyzed, according to the RNA-seq data from normal tissues of 95 human individuals. **b** qRT-PCR was performed in five CRC cell lines HCT116, SW620, SW480, HT29, DLD-1and 2 human adjacent non-cancer tissues. The relative mRNA levels are analyzed. The results represent at least three independent experiments and are reported as mean ± SEM. *p < 0.05

According to the RNA-seq data from normal tissues of 95 human individuals [12], *GAP43* gene is biasedly expressed in brain, adrenal, gall bladder, heart, placenta, prostate, salivary gland and a series of indigestive tissues, including duodenum, small intestine, colon and appendix (Fig. 1a). As previous observation of the downregulated *GAP43* in CRC, we hypothesize that GAP43 may play role on the development of CRC. To investigate the expression level and mutations of GAP43 in CRC tissues, we analyzed the publicly available databases (cBioPortal, Oncomine). In cBioPortal database, we found that GAP43 was mutated in CRC cancer tissues with the 1–3% frequency (Supplementary Fig. S1b), and in Oncomine, compared with normal colorectal tissues, we found that the expression of GAP43 was significantly repressed in CRC tissues (Supplementary Fig. S1c). Further validations were performed with Quantitative real-time PCR (qRT-PCR) in 3 paired CRC tissues and the corresponding adjacent non-tumor tissues, 1 normal colorectal epithelial cell line NCM460 and 5 CRC cell lines HCT116, SW620, SW480, HT29, DLD-1 (Fig. 1b). The results showed that GAP43 was significantly down-regulated in these 3 CRC tissues and 5 CRC cell lines, compared to normal tissues and normal colon cell, respectively. Thus, these data indicated that *GAP43* gene was expressed in human normal colon tissues, but significantly down-regulated in CRC.

### GAP43 level is repressed in CRC due to aberrant promoter methylation

To investigate the mechanisms involved in the silencing of *GAP43* gene in CRC, we investigated the DNA methylation and mRNA expression data of GAP43 from CRC tissue of the TCGA database. The results showed that the downregulated expression of GAP43 may due to the hyper DNA methylation of its promoter regions in CRC tissues (Supplementary Fig. S2).

**Fig. 2:**
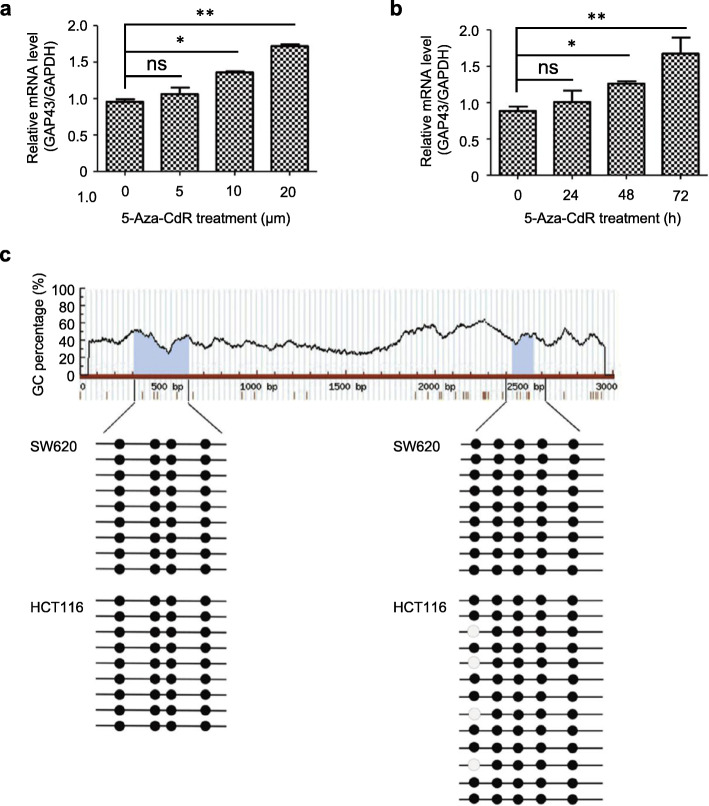
Silencing of *GAP43* gene is due to aberrant promoter methylation. **a** SW620 cells were treated with the indicated concentration of DNA demethylating agent 5-Aza-CdRfor 72 h, or **b** SW620 cells were treated with 10 μM 5-Aza-CdR for indicated treatment time (0, 24, 48, 72 h), and then mRNA levels of GAP43 were analyzed by qRT-PCR. **c** Bisulfite genomic sequencing was performed to analyze the methylation status of the GAP43 promoter in CRC cell lines SW620 and HCT116. Multiple CpG islands in the promoter region of the *GAP43* gene were analyzed. The results represent at least three independent experiments and are reported as mean ± SEM. *p < 0.05, **p < 0.001

To demonstrate that silencing of GAP43 expression in CRC cells is caused by the methylation of its promoter, we treated SW620 cells with the DNA demethylating agent 5-Aza-CdR. As shown in Fig. 2a, mRNA level of GAP43 was significantly increased after the treatment of SW620 cells with high concentrations of 5-Aza-CdR for 72 h. When SW620 cells were treated with 10 μM 5-Aza-CdR, the mRNA level of GAP43 was significantly increased with the increasing treatment time (Fig. 2b). To further confirm the DNA methylation in the GAP43 promoter of CRC cells, we performed bisulfite genomic sequencing to analyze the methylation status of the GAP43 promoter in CRC cell lines SW620 and HCT116. The promoters of HAND1 M1 and HAND1 M2 have been known to be highly methylated [13], and the promoters of ABCC1 and ABCG2 are rarely methylated [14]. Thereby, we take them as positive or negative controls in bisulfite genomic sequencing analysis, respectively (Fig. S3). The results showed that in the CRC, multiple CpG islands in the promoter region of the *GAP43* gene were methylated (Fig. 2c). These results show that the downregulation or silencing of GAP43 in CRC is associated with its promoter methylation.

### Identification of transcriptome alterations in GAP43 overexpressed CRC cells through RNA-seq

**Fig. 3:**
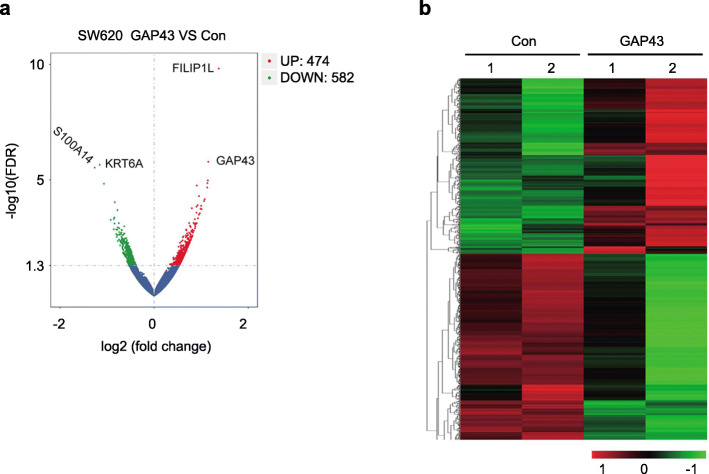
Heatmap and volcano plot. **a** The volcano plot was constructed by plotting the negative log of the log10 FDR value on the y-axis. These results in data points with low log10 FDR values (highly significant) appearing toward the top of the plot. The x-axis is the log (fold change) between GAP43-overexpressed and control SW620 cells. **b** The heatmap of up- and down-regulated genes in red and green, respectively

As GAP43 expression is silenced in CRC cells, in order to investigate the transcriptome alterations, we performed RNA-seq on GAP43-overexpressed SW620 cells and control SW620 cells. For transcriptome analysis, we acquired valid RNA-seq data from stable transfected GAP43 (29,592,934 bp and 25,180,042 bp) and control (28,506,301 bp and 27,436,701 bp) SW620 cells with Q30 > 90%, respectively (Supplementary Table S1). For more accurate results, the RNA-seq data was filtered with estimated FPKM values less than 1.0. Ultimately, with statistical analysis (Volcano plot), we found that the transcriptome of stable transfected GAP43 were significantly different from control SW620 cells (Fig. 3a). Totally 1056 differentially expressed genes (DEGs) were identified in stable transfected GAP43 compared with control SW620 cells. Among these genes, 474 genes were upregulated and 582 genes were downregulated, including upregulated FILP1L (WNT suppressor) [15], downregulated KRT6A (wound healing and epithelial migration) [16] and S100A14 (cell proliferation and cell migration) [17–20], et al. (Supplementary Table S2) (*p*-value< 0.05). The heatmap for some significantly altered genes by overexpressed GAP43 was shown in Fig. 3b. According to cellular functional categorization, significantly upregulated genes were classified as follows: transport (*p*-value = 2.60*E-04); glucose homeostasis (*p*-value = 5.51*E-04); retinoid metabolic process (*p*-value = 8.78*E-04); peripheral nervous system development (*p*-value = 8.93*E-04); extracellular matrix organization (*p*-value = 1.05*E-03). The significantly downregulated genes were classified as follows: translation (*p*-value = 8.02*E-56); SRP-dependent cotranslational protein, targeting to membrane (*p*-value = 2.98*E-54); nuclear-transcribed mRNA catabolic process, nonsense-mediated decay (*p*-value = 3.62*E-50); rRNA processing (*p*-value = 1.92*E-49); translational initiation (*p*-value = 7.24*E-49) (Supplementary Table S3).

### Transcriptome analyses of GAP43-overexpressed CRC cells through DAVID and IPA

**Fig. 4:**
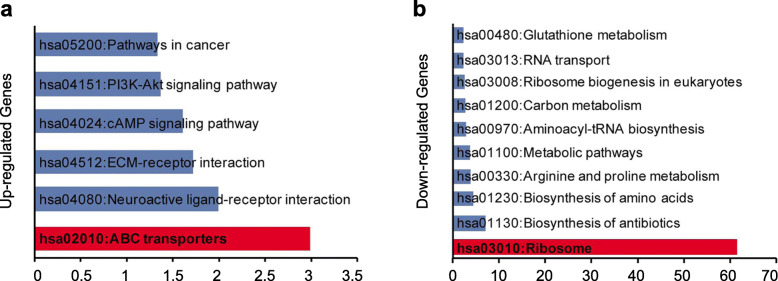
Functional pathway enrichment analysis. **a** The upregulated DEGs or **b** down-regulated DEGs are involved in various KEGG biological pathways

After bioinformatics analysis of RNA-seq data, the upregulated or downregulated genes in GAP43 stable transfected SW620 cells were summarized. We submitted these genes to DAVID (a web-based high-throughput functional genomics analysis tool) for systematically clustering these genes. In GOTERM_BP_FAT category, we identified significantly enriched GO terms that characterized GAP43 stable transfected SW620 cells. In total, 24 subcategories (*p*-value< 0.01) were found among upregulated genes and 38 subcategories (*p*-value< 0.01) among downregulated genes (Supplementary Table S3). Among the up-regulated genes, we found that cell membrane proteins were significantly enriched, such as transport (*p*-value = 2.60*E-04) and extracellular matrix organization (*p*-value = 0.001); while among down-regulated genes, protein translation related genes were significantly enriched. In KEGG_PATHWAY category, we found that the upregulated genes were clustered into 6 subcategories (*p*-value< 0.05, 1 subcategories with *p*-value< 0.01), including ABC transporters (*p*-value = 0.001) and ECM-receptor interaction (*p*-value = 0.019) (Fig. 4a). While the down-regulated genes were clustered into 12 subcategories (*p*-value< 0.05, 10 subcategories with *p*-value< 0.01) (Fig. 4b), including ribosome (*p*-value = 2.98*E-62); biosynthesis of antibiotics (*p*-value = 6.87*E-08); biosynthesis of amino acids (*p*-value = 3.11*E-05); arginine and proline metabolism (*p*-value = 1.35*E-04); metabolic pathways (*p*-value = 1.61*E-04) (Supplementary Table S4). These results showed that overexpressed GAP43 may influence the membrane transporters, and then repress the cancer cells’ metabolism and cause the repression of proliferation, invasion and migration effects in CRC cells.

**Fig. 5:**
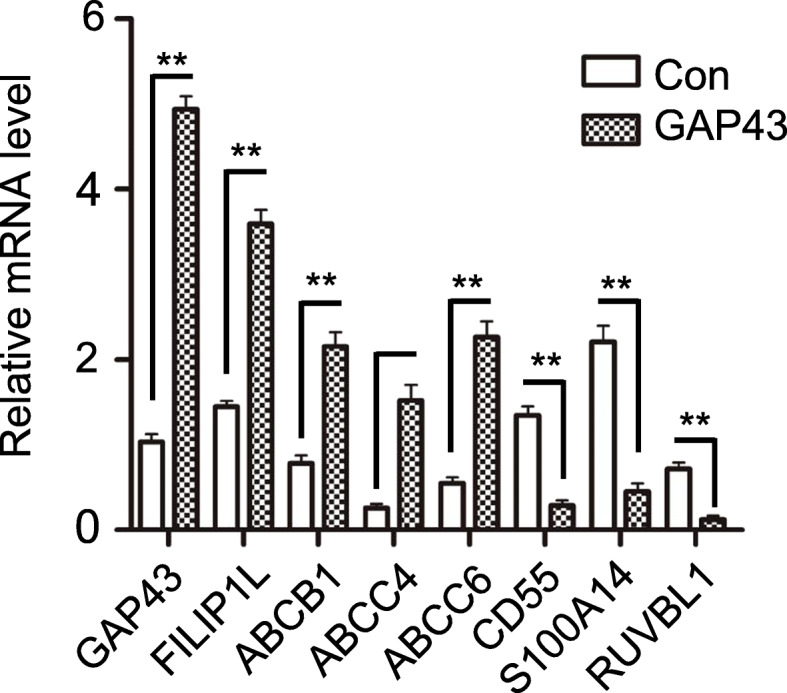
The expression levels of the most significant DEGs in RNA-seq data were confirmed by qRT-PCR in GAP43-overexpressed and control SW620 cells

**Fig. 6:**
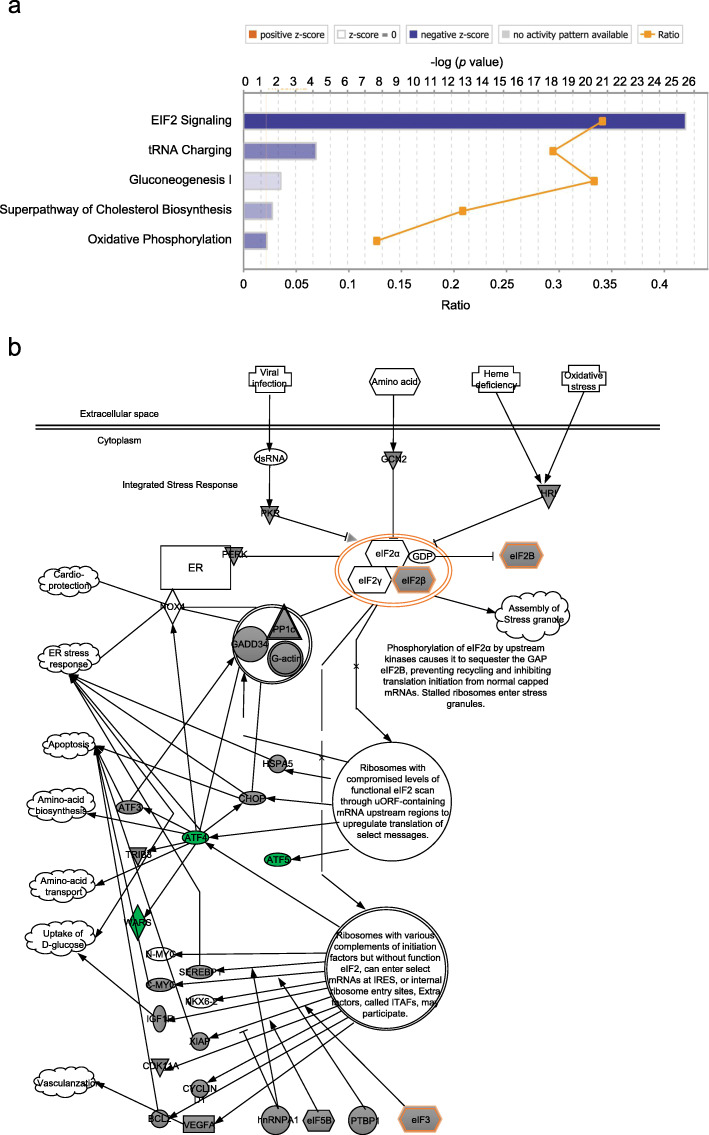
IPA analysis and EIF2 signaling pathway. **a** IPA analysis. **b** EIF2 signaling pathway

The expression levels of the most significant DEGs in RNA-seq data were confirmed by qRT-PCR in GAP43-overexpressed and control SW620 cells (Fig. 5). Furthermore, we applied these DEGs into IPA analysis, and we found that the most significant is the EIF2 signaling pathway (−log(*p*-value) = 25.5 and z-score = − 4.802) (Fig. 6a) [21, 22]. In EIF2 signaling pathway, the translation initiation factor (eIF1) and transcriptional factor (ATF4 and ATF5) were significantly downregulated (Fig. 6b). These results suggested that except the function on membrane, GAP43 may also involve in the regulation of cellular translation through the EIF2 signaling pathway.

## Discussion

In previous studies, the functional studies of GAP43 were focused on the neuronal development, and the protein was considered to play crucial roles in effective regenerative responses, and learning and memory processes of the nervous system [23–25]. GAP43 is highly expressed in nervous system, and also biasedly expressed in indigestive tissues, including duodenum, small intestine, colon and appendix. Recent study has shown that GAP43 serves as a specific biomarker of relapsed or refractory neuroblastoma [26, 27]. However, the relationship between GAP43 and CRC development has been uncovered. In this study, we confirmed that GAP43 is expressed in human colon tissues, but is downregulated in CRC cells and tissues due to the methylation of its promoter region.

Aberrant epigenetic changes frequently occur in human colorectal cancer. The accumulation of numerous epigenetic alterations may drive the initiation and progression through the adenoma-carcinoma sequence [28, 29]. We found the expression pattern of GAP43 in colorectal cancer cells was closely associated with cancer-linked DNA hypermethylation, and the methylation status of GAP43 promoter did not change in different colorectal cancer cell lines (Fig. 2c). Additionally, we found that the expression level of GAP43 in immortalized colorectal epithelial cell line NCM460 was significantly higher than those in CRC cell lines, but it was dramatically reduced compared to normal colorectal tissues (Fig. 1b). Thus, when comparing the *GAP43* gene expression patterns in a series of CRC cell lines, immortalized colorectal epithelial cell line, tumor cells and normal cells, we deduced that the accumulation of aberrant methylation of *GAP43* gene might drive the initiation of CRC.

Further transcriptome analysis indicated multiple potential biological functions of GAP43 in CRC. The most significant different expression gene is upregulated *FILIP1L*, downregulated *KRT6A* and *S100A14*. While FILIP1L is a WNT pathway inhibitor [15], and the reduction of *FILIP1L* in ovarian cancer is associated with poor survival, progression and chemoresistance [30]. Previous studies showed that KRT6A and S100A14 were involved in cell migration and cell proliferation [16, 17, 20]. These results remind us that the reduced level of GAP43 in CRC due to its transcriptional alterations might regulate some potential signaling pathways associated with tumorigenesis, such as WNT signaling.

In KEGG_PATHWAY category, we found that the upregulated genes were clustered into 6 subcategories including ATP-binding cassette (ABC) transporters (*p* = 0.001). ABC transporters constitute a superfamily of membrane proteins that couple the hydrolysis of adenosine triphosphate (ATP) to vectorial transport of substrates across biological membranes [31]. Previous studies have showed that ABC superfamily utilizes the free-energy from ATP hydrolysis to shuttle many different substrates across various biological membranes, and consequently, are involved in both normal and abnormal physiology [32]. In this study, we found that with the overexpression of GAP43 in CRC cells, the expression of ABC transporters is also upregulated. However, the expression of bothGAP43 and ABC transporters is down-regulated in CRC tissues and cell lines, which indicates that ABC transporters might be related with the development of CRC.

In addition, we analyzed the transcriptome of GAP43 overexpressed CRC cells through DAVID and IPA. The IPA analysis showed that EIF2 signaling pathway is the most significant psychological phenomena (−log(*p*-value) =25.5 and z-score = − 4.802) (Fig. 6a, Supplementary Table S5), and three important members (eIF1, ATF4, ATF5) of EIF2 signaling pathway were significantly downregulated (Fig. 6b). Incidentally, we also found that GAP43 is differentially expressed, elevated or decreased in gastric cancer. Survival analysis showed that there was no significant change in survival of gastric cancer patients with low or high expression of GAP43 (Fig. S4). However, we found that patients with gastric cancer who were treated with the 5-FU anticancer drug had higher survival rates in patients with high GAP43 expression than those with low expression (Fig. S4). This suggests that GAP43 is likely to be associated with drug resistance to cancer chemotherapy drugs. Considering that the tolerance of chemotherapy drugs is related with ABC transporters, and GAP43 level is associated with the expression of ABC transporters. Therefore, GAP43 might affect the chemotherapy drug resistance in cancer patients through ABC transporter-mediated signaling pathway.

## Conclusions

GAP43 is expressed in human indigestive tissues, including colon tissues, but is downregulated in CRC due to the methylation of its promoter region. The RNA-Seq transcriptome data analysis showed the gene expression profile between GAP43-overexpressed and control CRC cells. The analysis of GAP43-overexpressed versus control CRC cell line data gives insight into potential genes and pathways that may uncover the functional roles of GAP43 in the development of human CRC and expand the knowledge of the CRC progression.

## Supplementary Information

**Additional file 1: Supplementary Figure S1.** Mutation and expression level analysis of GAP43 in CRC tissues with online database. a Expression of GAP43 in CRC (COAD and READ). b Mutation level of GAP43 in CRC tissues with cBioPortal database. **c** Expression level of GAP43 in CRC tissues with Oncomine database.

**Additional file 2: Supplementary Figure S2.** Correlation analysis of GAP43 expression level and its promoter methylation level in colon adenocarcinoma (a) and rectal adenocarcinoma (b).

**Additional file 3: Supplementary Figure S3.** Bisulfite genomic sequencing was performed to analyze the methylation status of the ABCC1 (a), ABCG2 (b), HAND1 M1 (c) and HAND1 M2 (d) promoters in CRC cell line HCT116. Multiple CpG islands in the promoter regions were analyzed.

**Additional file 4: Supplementary Figure S4.** Kaplan-Meier Plotter survival analysis of GAP43 in gastric cancer with or without different treatments. a gastric cancer. b gastric cancer with surgery only. c gastric cancer with 5-FU based adjuvant.

**Additional file 5: Supplementary Table S1.** Quality Control (QC) of our RNA-seq data.

**Additional file 6: Supplementary Table S2.** GAP43 overexpression related genes revealed by RNA-seq. (*p*-value< 0.05).

**Additional file 7: Supplementary Table S3.** DAVID analysis (GO_BP) of GAP43 overexpression related genes (*p*-value < 0.05).

**Additional file 8: Supplementary Table S4.** DAVID analysis (KEGG) of GAP43 overexpression related genes (*p*-value < 0.05).

**Additional file 9: Supplementary Table S5.** IPA Canonical Pathways of GAP43 overexpression related genes.

**Additional file 10: Supplementary Table S6.** Clinical Information of three CRC samples used in expression analysis of GAP43.

**Additional file 11: Supplementary Table S7.** Primers for qRT-PCR.

## Data Availability

All data generated or analyzed during this study are included in this published article. The RNA-seq datasets analyzed in the present study are available from the GEO repository, GSE145180 (http://www.ncbi.nlm.nih.gov/geo/query/acc.cgi?acc=GSE145 180).

## Abbreviations

GAP43: Growth associated protein 43
ABC: ATP-binding cassette transporter
EIF2: Eukaryotic initiation factor 2
CRC: Colorectal cancer
COAD: Colon adenocarcinoma
IPA: Ingenuity pathway analysis
ECM: Extracellular matrix
5-AZA-CdR: 5-AZA-2′-deoxycytidine
PI3K: Phosphoinositide 3-kinases
GPCR: G protein-coupled receptor
FGFR: Fibroblast growth factor receptor
MAPK: Mitogen-activated protein kinase
TGFB: Transforming growth factor beta
FILIP1L: Filamin A Interacting Protein 1 Like
KRT6A: Keratin 6A
ATF: Activating Transcription Factor
S100A14: S100 Calcium Binding Protein A14
ATP: Adenosine triphosphate
DEGs: Differentially expressed genes
GO: Gene Ontology
KEGG: Kyoto Encyclopedia of Genes and Genomes
FPKM: Fragments per kilobase of exon model per million reads mapped
GEPIA: Gene Expression Profiling Interactive Analysis
DAVID: Database for Annotation, Visualization and Integrated Discovery
