# Supplementary figures and images for: Transcriptome profiling unveils GAP43 regulates ABC transporters and EIF2 signaling in colorectal cancer cells
Source: BMC Cancer. 2021 Jan 5;21:24. doi: 10.1186/s12885-020-07728-x (PMC7786480; doi:10.1186/s12885-020-07728-x)

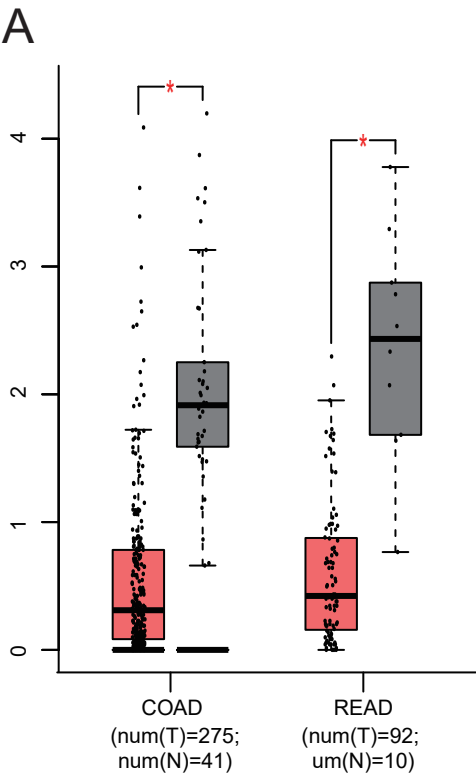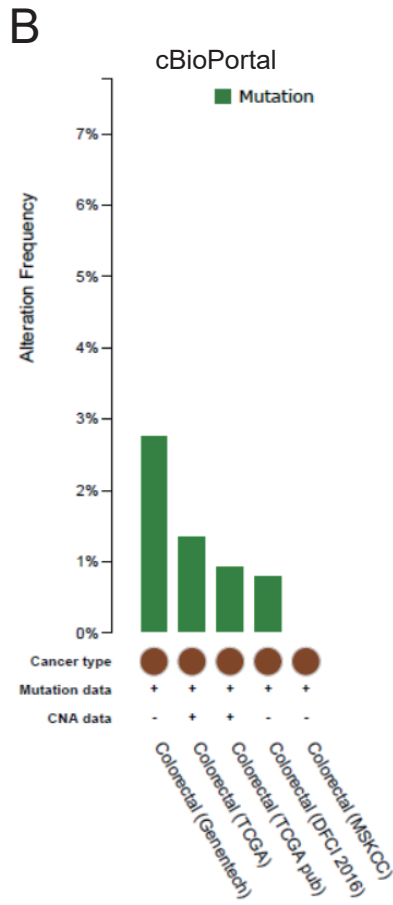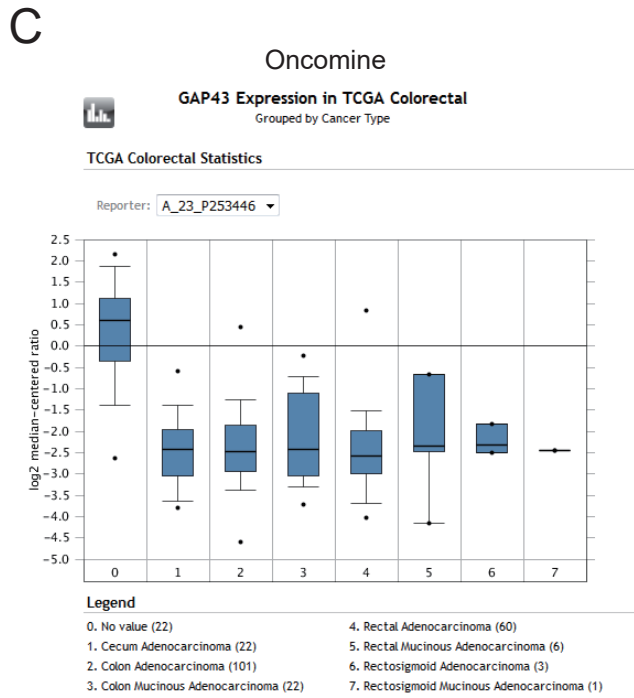

Supplement: Supplementary file 1 — Additional file 1: Supplementary Figure S1. Mutation and expression level analysis of GAP43 in CRC tissues with online database. a Expression of GAP43 in CRC (COAD and READ). b Mutation level of GAP43 in CRC tissues with cBioPortal database. c Expression level of GAP43 in CRC tissues with Oncomine database. [file 12885_2020_7728_MOESM1_ESM.pdf]

A

colon\_adenocarcinoma

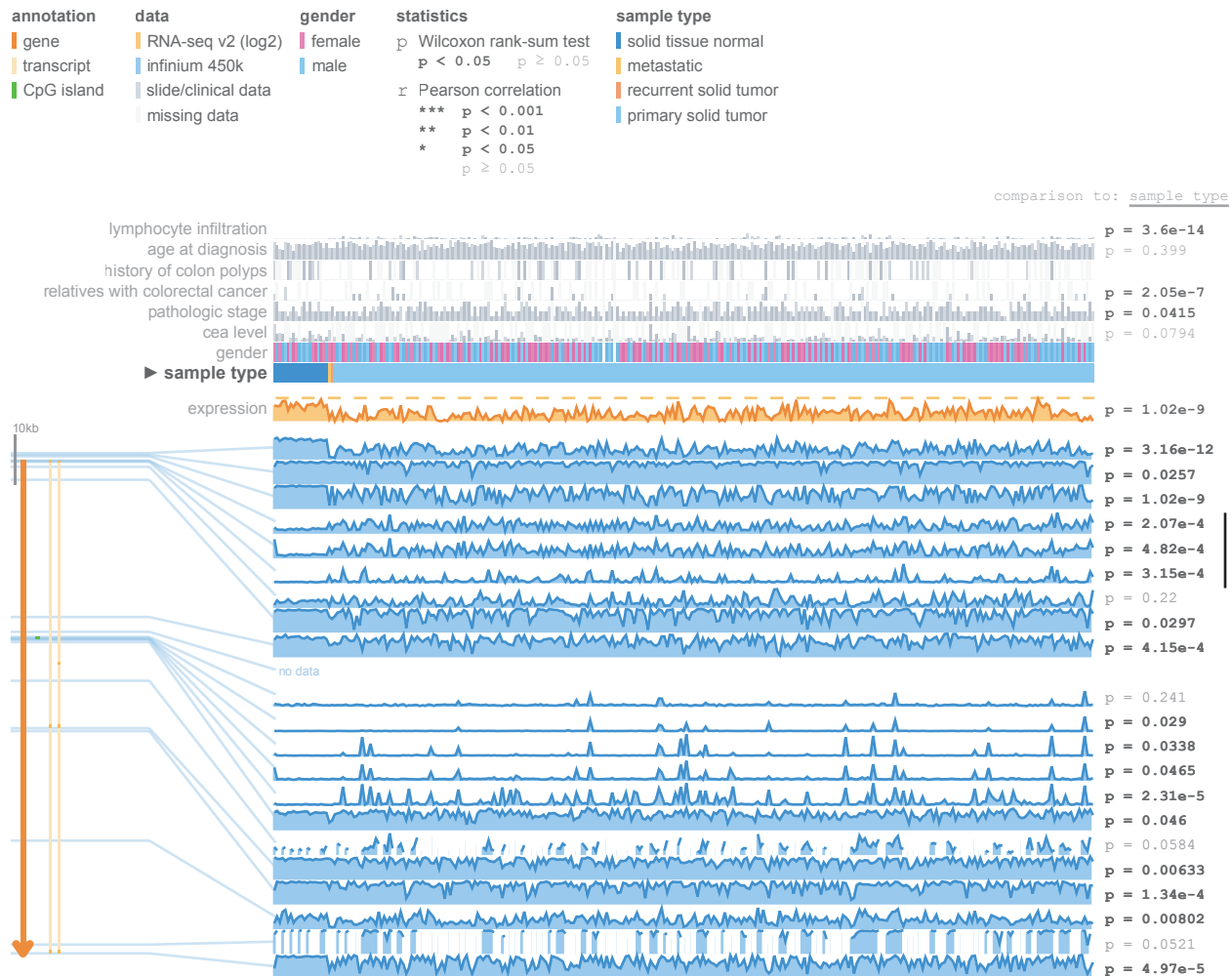

B

colorectal\_adenocarcinoma

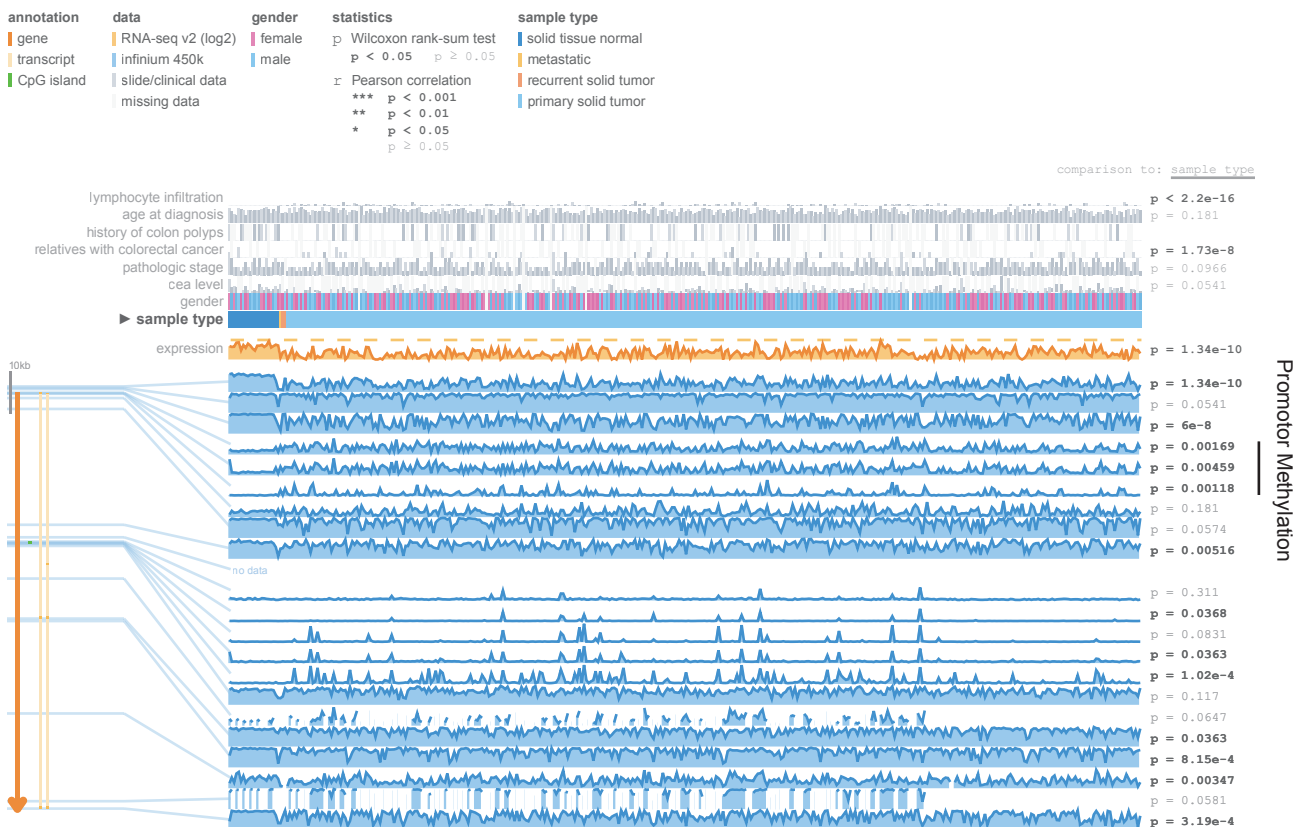

Supplement: Supplementary file 2 — Additional file 2: Supplementary Figure S2. Correlation analysis of GAP43 expression level and its promoter methylation level in colon adenocarcinoma (a) and rectal adenocarcinoma (b). [file 12885_2020_7728_MOESM2_ESM.pdf]

**A**

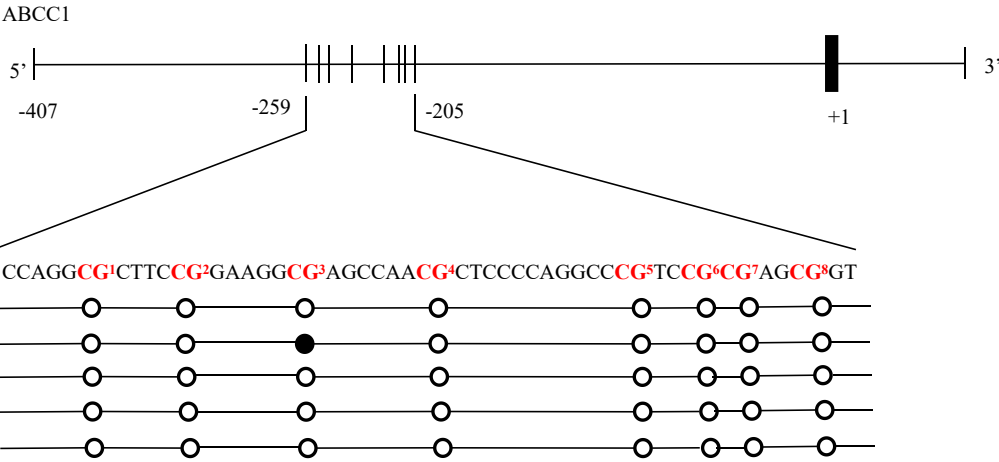

**B**

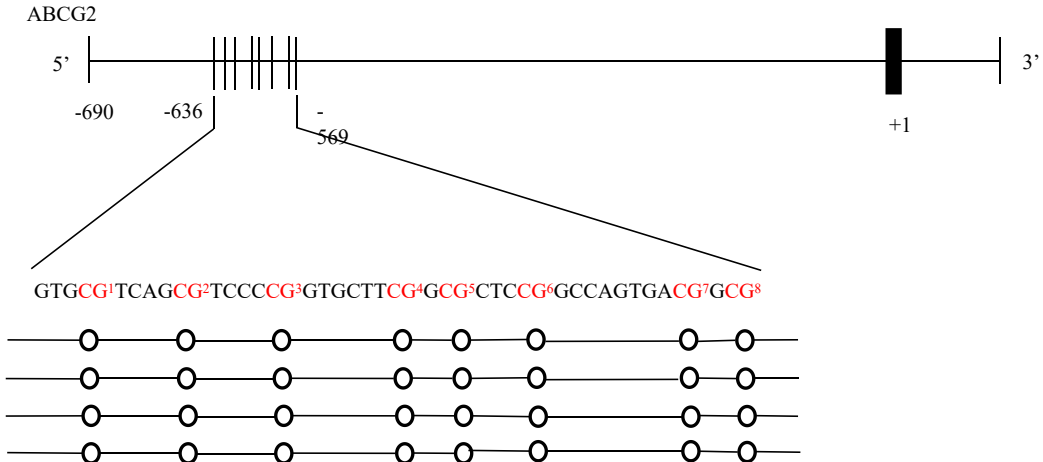

**C**

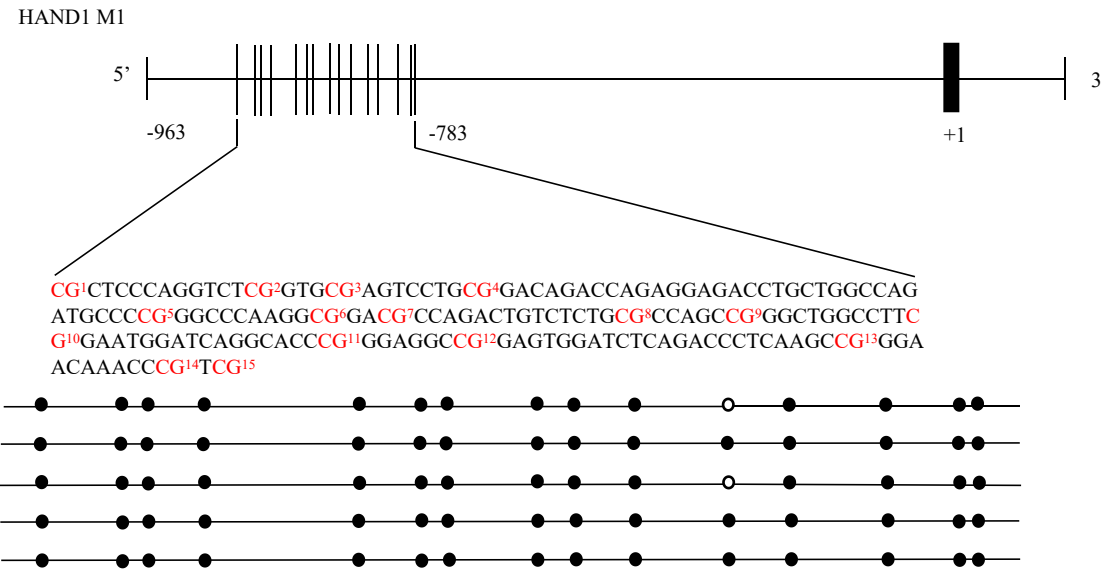

**D**

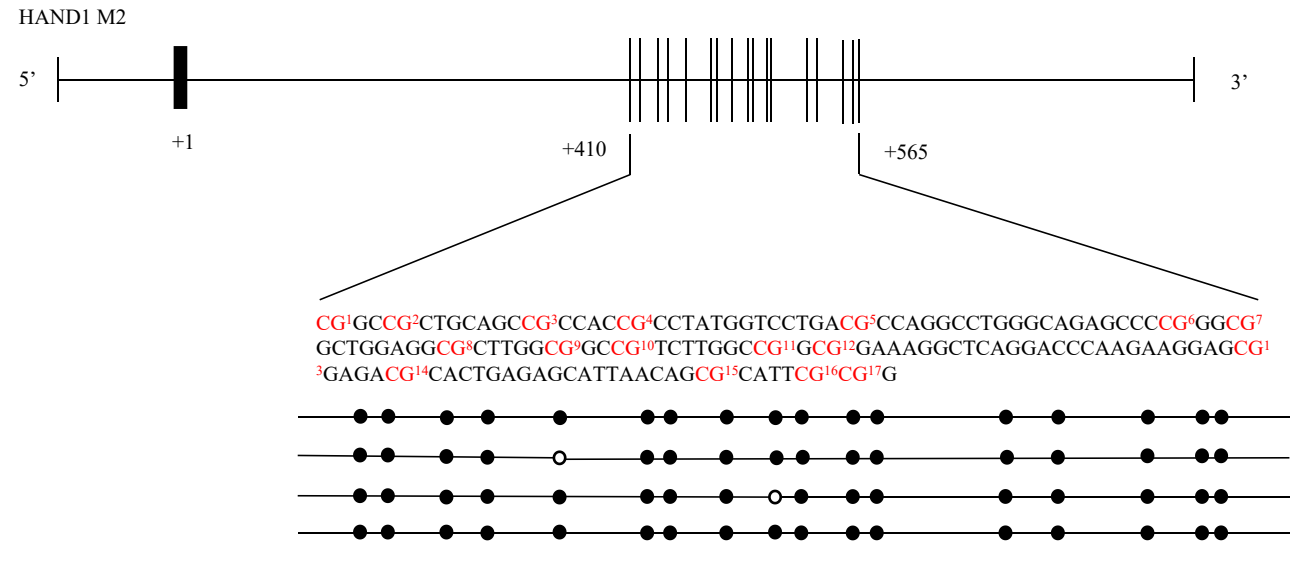

Supplement: Supplementary file 3 — Additional file 3: Supplementary Figure S3. Bisulfite genomic sequencing was performed to analyze the methylation status of the ABCC1 (a), ABCG2 (b), HAND1 M1 (c) and HAND1 M2 (d) promoters in CRC cell line HCT116. Multiple CpG islands in the promoter regions were analyzed. [file 12885_2020_7728_MOESM3_ESM.pdf]

A

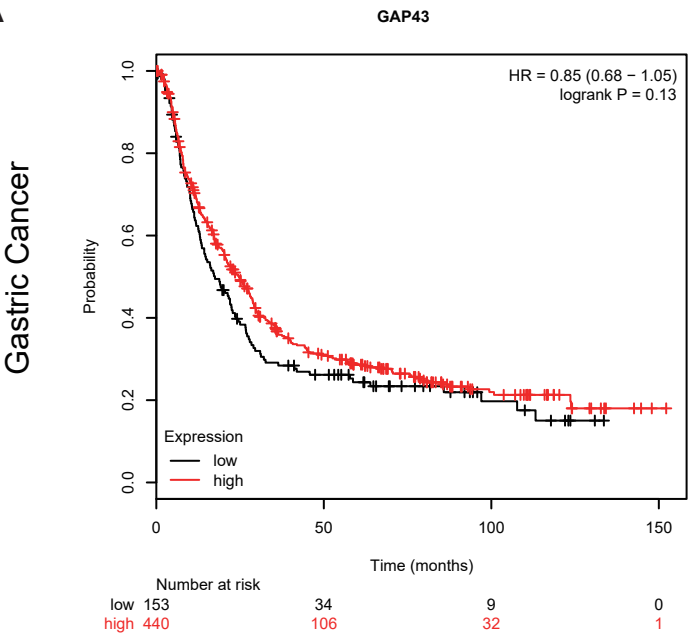

B

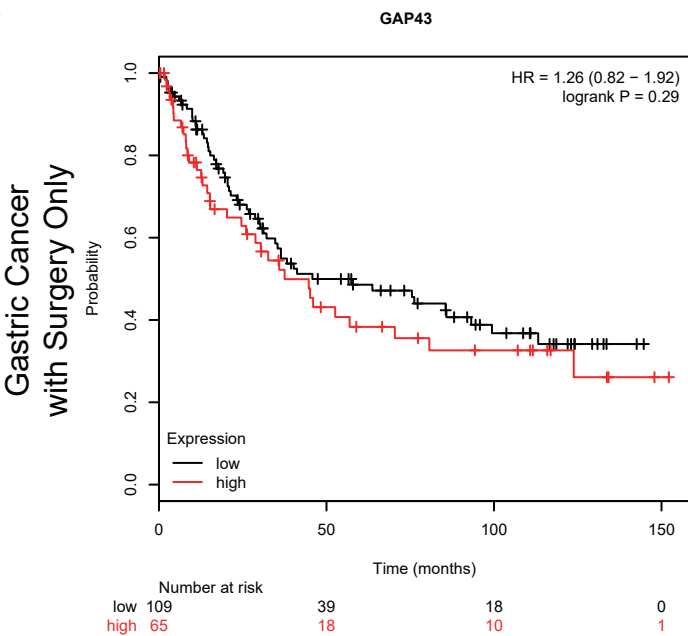

C

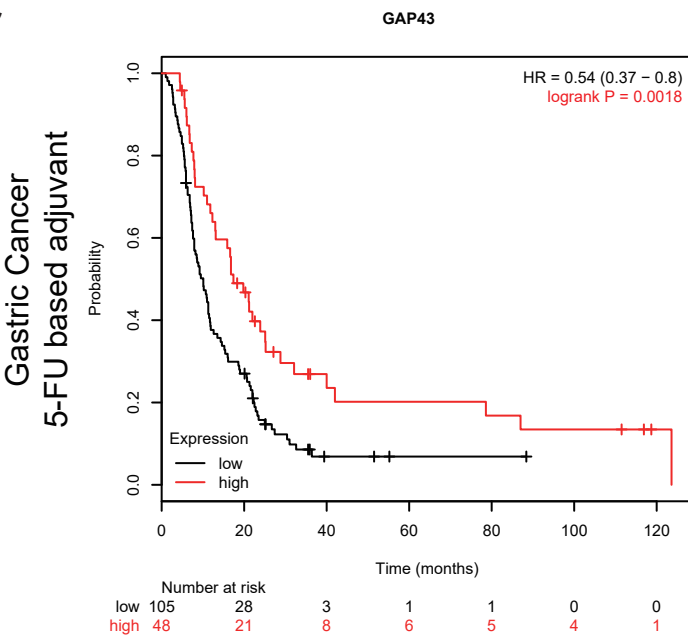

Supplement: Supplementary file 4 — Additional file 4: Supplementary Figure S4. Kaplan-Meier Plotter survival analysis of GAP43 in gastric cancer with or without different treatments. a gastric cancer. b gastric cancer with surgery only. c gastric cancer with 5-FU based adjuvant. [file 12885_2020_7728_MOESM4_ESM.pdf]
